# EXPLORING CONTEMPORARY ISSUES IMPACTING OLDER REFUGEES IN THE UNITED STATES

**DOI:** 10.1093/geroni/igae098.0766

**Published:** 2024-12-31

**Authors:** Noelle Fields, Jonix Owino

**Affiliations:** Chair; Discussant

## Abstract

Older refugees represent a unique group of the immigrant population as they are often at higher risk for negative health and psychosocial outcomes. Many older refugees also face constraints posed by language and cultural barriers that can affect overall well-being and quality of life. This symposium will provide insights from three studies of older refugees in the United States in an effort to shed light on various issues impacting this population that warrant greater attention from researchers and practitioners. The first paper presents findings from a study of older immigrants and refugees’ preparation for weather-related emergencies. Findings suggest the importance of linguistically and culturally tailored communication that fosters trust and improved access to reliable information. The second paper explores the lived experiences of forced displacement and long-term resettlement of Karen refugees from Burma. Study findings point toward the need to re-evaluate the cross-cultural validity of locus of control, an oft used psychological measure applied to refugee populations. The third paper examines how socioeconomic status is differentially associated with cognitive difficulties between older Bhutanese refugees and other refugee groups. Study results suggest that higher income does not serve as a protective factor against cognitive challenges in older refugees from Bhutan. The symposium will conclude with a critical reflection on the empirical and theoretical contributions needed to advance scholarship on older refugees.

